# “A rare case of bladder myiasis mimicking radiation cystitis—A first case report”

**DOI:** 10.1002/ccr3.9240

**Published:** 2024-08-06

**Authors:** Mahendra Kumar, Sujit Saikia, Anup Kumar Das, Debabrata Barmon, Upasana Baruah, Dimpy Begum, Sopouassi V. Nicholas King

**Affiliations:** ^1^ Department of Gynaecological Oncology Dr. B. Borooah Cancer Institute Guwahati Assam India; ^2^ Department of Urology Central Nursing Home Guwahati Assam India; ^3^ Department of Oncopathology Arya Wellness Centre Guwahati Assam India

**Keywords:** bladder myiasis, complications in cancer survivors, endometrial cancer, gynecological cancer, post‐radiation cystitis, sanitation awareness

## Abstract

Patients diagnosed with cancer post‐treatment are prone to have recurrent disease. Regular follow‐up of these patients enables early recognition and treatment. A tissue diagnosis before starting treatment is imperative to avoid misdiagnosis and management. Given their immunosuppression, maintaining good nutrition, body hygiene, and clean surroundings is essential to prevent the most common urinary tract infection to rare urinary myiasis infection. *Cochliomyia hominivorax*, *Chrysomya bezziana*, and *Wohlfahrtia magnifica* are the most prevalent flies causing myiasis in human beings. Risk factors for urinary myiasis are open or untreated wounds and debris around the genital area. Specific flies, ticks, and mosquitoes are responsible for myiasis in tropical or subtropical countries, when it is usually not transmitted from human to human. Flies lay their eggs near the urethra, and the larvae hatch and migrate to the bladder. Increased urinary frequency, haematuria, and lower abdominal pain are the most commonly found presenting symptoms. Simple preventive measures can avoid these complications. So, prevention is better than cure.

## 1 INTRODUCTION

Regular and diligent follow‐up post‐treatment is not just necessary; it is paramount with the advancements in the treatment of various gynecology cancers. It aids in the early detection of recurrences and the management of side effects of primary therapy. Imaging techniques such as contrast‐enhanced computed tomography (CECT) are often employed to detect recurrences. However, obtaining a tissue diagnosis before confirming a recurrence is crucial. Tissue biopsy enables an accurate diagnosis, thereby preventing the potential adverse effects of chemotherapy and radiation. Tissue biopsy underscores the urgency and significance of regular follow‐up in gynecological cancer patients.

Cancer and its treatment can lead to a weakened immune system and weight loss, making it imperative for cancer survivors to maintain a healthy diet, good hygiene, and regular exercise. Failure to do so can increase the risk of infections, weight loss, and recurrences.^1^ The immune system is our body's defense against infections. However, cancer and chemotherapy can compromise this system by reducing the number of infection‐fighting white blood cells. It is important to note that immunodeficiency is a significant factor in cancer development, and infection can also lead to immunodeficiency. The risk of malignancy increases with severe primary immunodeficiency.^2^

Increased incidences of urinary tract infections are seen in cancer survivor patients, especially in gynecological cancer, due to debris present in the vagina after radiation in cervical cancer, post‐radiation vaginal dryness, and post‐radiation genital infection. Endometrial cancer survivors had higher risks of genitourinary outcomes compared to a general population of women.^3^ Urinary and genital tract infections can be of various types, with bladder myiasis being one of the rarest types. The most common presentation of bladder myiasis is urinary tract infections, haematuria, and lower abdominal pain.^4^ These symptoms mimic other common etiology, and diagnosing this rare becomes more difficult. It underscores the potential complications and the need for caution in such cases, highlighting the gravity and seriousness of the situation.

We present an intriguing and exceedingly rare case of bladder myiasis, a condition that mimics radiation cystitis, diagnosed during the follow‐up of a post‐radiation endometrial cancer patient. This unique and sporadic case, which is the first of its kind, underscores the importance of vigilance in post‐treatment follow‐up and the potential complications that can arise, even in the most uncommon scenarios.

## 2 CASE REPORT

### 2.1 Case history

A 51‐year‐old postmenopausal lady, a homemaker belonging to the lower middle class of socioeconomic status, by modified Kuppuswami scale, was referred to our tertiary care institute. She had undergone total abdominal hysterectomy with bilateral salpingo‐opherectomy in 2015, given postmenopausal bleeding and biopsy report suggestive of papillary endometrioid endometrial carcinoma. A review of the biopsy report showed papillary endometrioid carcinoma of the endometrium with less than 50% myometrial invasion and Lymph vascular space invasion (LVSI) involvement. On discussion in the multidisciplinary tumor board, adjuvant radiation with external beam radiotherapy because of the high intermediate risk group (LVSI positive) was taken. The patient had completed radiation (EBRT—50Gy in 25# and CVS 7Gy in 3#) till February 2016, following which she was on regular follow‐up. During follow‐up after a disease‐free interval of 58 months, she had a pelvic recurrence in December 2020, and the CECT scan detected a 3.7 × 1.7 cm irregular mass in the pelvis, considered as recurrence (biopsy proven). The multidisciplinary tumor board decided on palliative radiotherapy. She had received 30Gy in 10# between 19/1/2021 and 02/02/2021 and, after completion of treatment, kept on regular follow‐up.

### 2.2 Present illness and management

In December 2021, the patient had mild, intermittent, and scanty haematuria. There was no history of fever, chills, or rigor. On follow‐up, the patient performed a blood investigation and urine examination as a routine check‐up. Microscopic urine examination showed few pus cells and RBCs. USG KUB suggested mild cystitis and significant residual urine volume in the urinary bladder. Cystoscopy showed blood clots with multiple telangiectasias, predominantly on the dome of the bladder. The oncologist managed the patient conservatively, and the symptoms resolved during this period.

**FIGURE 1:**
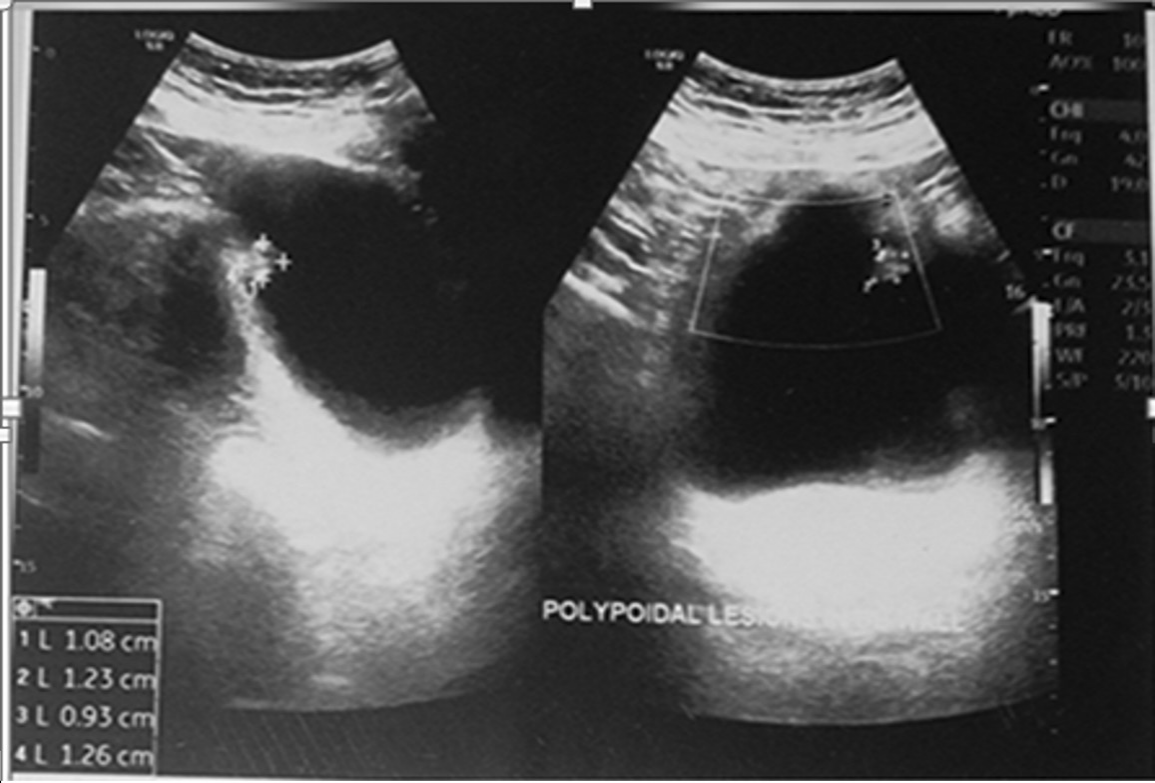
Transabdominal pelvic ultrasound showing urinary bladder, debris floating in urine with thickened urinary bladder wall (signs of cystitis) with a polypoidal lesion in the posterior wall of the urinary bladder (boundary marked with +).

**FIGURE 2:**
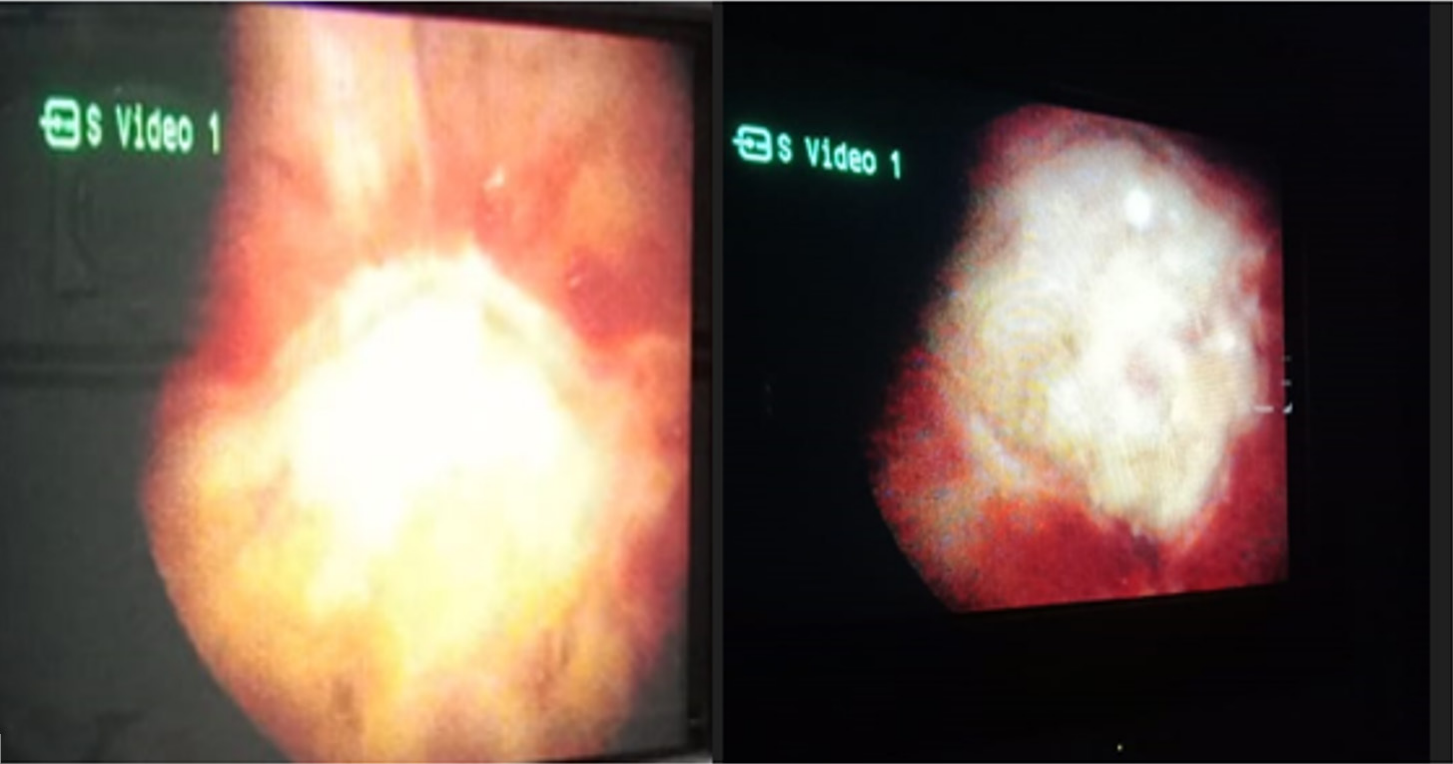
Cystoscopic view of the fundus region of the urinary bladder, showing macroscopic mucosal growth with surrounding dilated blood vessels (telangiectasias) with debris.

**FIGURE 3:**
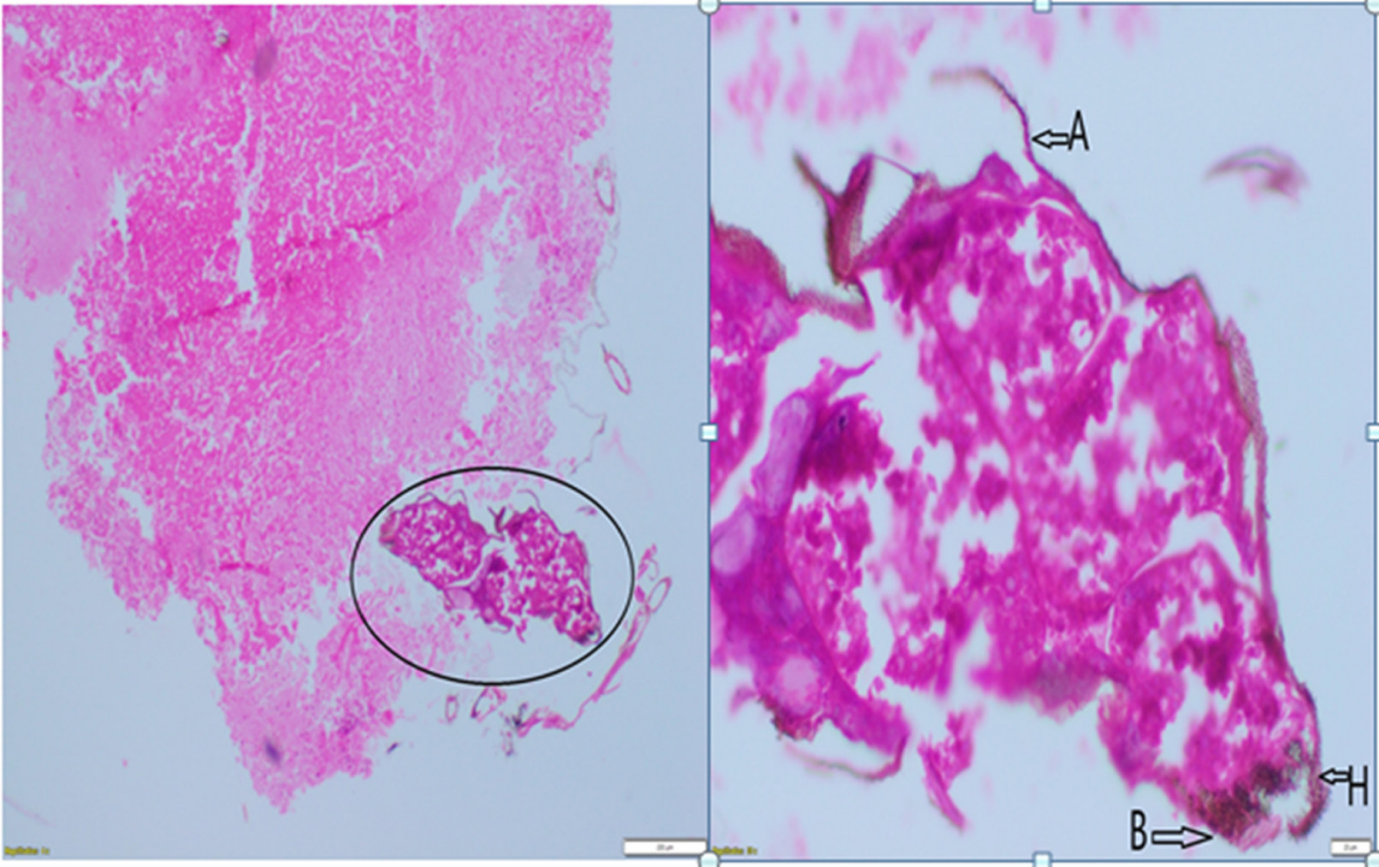
Shows an H&E‐stained slide of polypoidal lesions in the urinary bladder. Part 1 shows a larvae‐like structure under a low‐power field (encircling). Part 2 shows the head like structure of larvae, labeled as hook (H), mouth brushes (B), and appendages (A) under a high power field.

**FIGURE 4:**
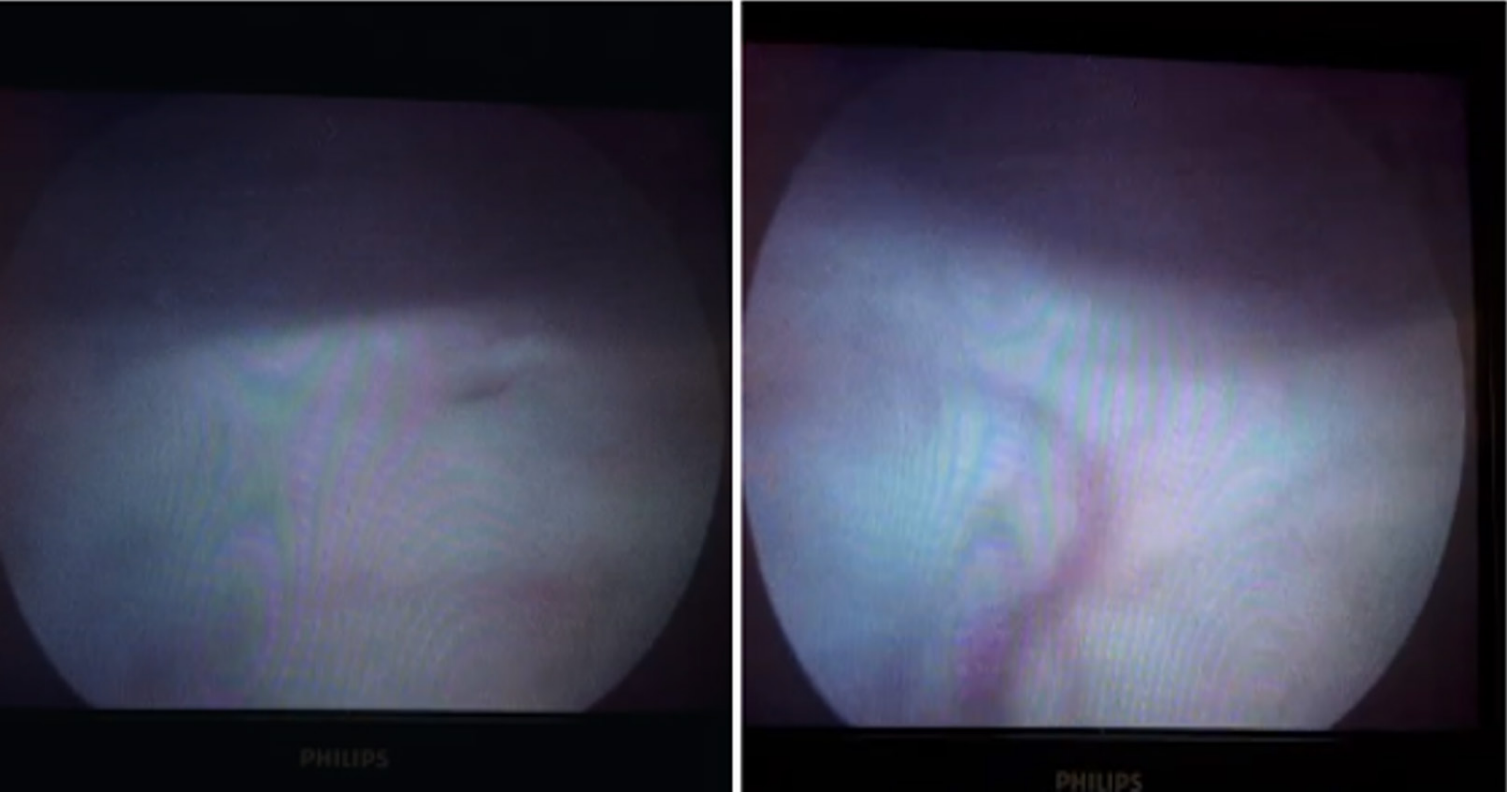
Cystoscopic view of the urinary bladder after recovery showing the healed area of urinary bladder growth with remaining telengactiasias.

After 6 months (July 2022), she came for a follow‐up visit with complaints of haematuria. USG done was suggestive of a small polypoidal lesion (12mmx10mm) at the dome of the urinary bladder (Figure 1); the findings in the urinary bladder were interpreted as a recurrence of the disease and referred to a urologist for cystoscopy and its management. Cystoscopy showed 12 × 10mm growth at the dome of the urinary bladder with surrounding telengactiasias (Figure 2). Cystoscopy‐guided biopsy of the mass done and histopathological examination suggested a maggot/larvae with no malignant tissue (Figure 3). Species of larvae could not identified. She received a single dose of Ivermectin 12 mg per oral and oral antibiotics for UTI.^5^ Repeat USG KUB was done after 1 month, which was expected to result in normal cystoscopy findings (Figure 4). The patient is on regular follow‐up now and is asymptomatic.

## 3 DISCUSSION

Human myiasis is reported in every part of the world and is prevalent mainly among lower socioeconomic status and global agricultural concerns when occurring in domestic animals. Myiasis is an injection of fly larvae into the human body, classified as cutaneous, gastrointestinal, nasopharyngeal, ocular, and urinary.^6^ Myiasis can be obligatory or facultative parasitosis. *Cochliomyia hominivorax*, *Chrysomya bezziana*, and *Wohlfahrtia magnifica* are the most prevalent flies causing obligatory human wounds worldwide.^6^ Cutaneous myiasis is the most common clinical form, followed by nasopharyngeal myiasis.^7^ Ocular myiasis and intestinal myiasis are other forms of myiasis due to ingestion of the organism, but genito‐urinary myiasis, constituting only 0.7%, is rarely reported.^8^, ^9^ A systemic review of human urogenital myiasis showing the vagina is the most common site in urogenital myiasis.^10^ Bladder myiasis is a rare, uncommon finding, even for an experienced urologist who performs cystoscopies.

Genitourinary myiasis involves the labia minora, urinary bladder, urethral meatus, and vaginal canal. Therefore, in bladder myiasis, the flies may lay their eggs near the urethra, and then, the larvae hatch and migrate to the bladder. Larvae of *Fannia scalaris* are the most common cause of bladder myiasis. Other fly genera, such as *Musca*, *Sarcophaga*, *Lucilia*, *Wohlfahrtia*, and *Calliphora*, are also associated with bladder myiasis.^10^

Urogenital discharges attract flies around the external genital and urethral orifice. The larva may have passed upwards through the urethra into the urinary bladder, causing cystitis, increased urination frequency, haematuria, and lower abdominal pain. Bladder myiasis patients may complain of urinary frequency, irritation, dysuria, and itching. Vomiting and side pain may also be observed.^7^, ^11^, ^12^

Literature has similar case reports in non‐cancer patients. Literature published a case report of a 20‐year‐old female of the lower socio‐economical class presented with complaints of the repeated passage of live worms in urine, dysuria, fever, and itching in the per urethral and genital regions on and off for the last few days. The patient performed a complete urine analysis and stool examination using direct and concentrated smears, and the smears showed larvae. The patient has undergone a cystoscopy. The bladder neck was congested with minor diffuse ulceration, and bladder wash fluid contained few larvae. The treating doctor counseled the patient to maintain hygiene and hydration daily with 3 liters of water. She received a single dose of Ivermectin 12 mg and a course of urinary antibiotics. The patient is asymptomatic, with no worms in urine.^11^

Literature published another case report of a young, sexually inactive female student who presented with necrotic growth in the paraurethral region infested with numerous maggots. The lesion involved the urethra and the bladder base. She received treatment through debridement of wound and bladder irrigation.^8^

A case report of a 31‐year‐old male who first presented with the hesitancy of urine, the passage of 2–3 larvae during urination, and a single episode of gross haematuria. The patient worked as a manufacturing laborer and described discontinuous housing and poor living conditions (unclean, damp environment, fly infestation) in the months preceding diagnosis. Direct examination of the initial urine specimen showed morphology consistent with free‐living fly larvae. The species is unknown. Due to the history of gross haematuria and a recent diagnosis of myiasis, the patient underwent a procedure of cystoscopy. After submitting urine and stool specimens, the patient received a single dose of oral Ivermectin 3 mg. The patient had improved living conditions and demonstrated an understanding that proper hygiene and sanitation may foster the prevention of recurrence.^13^

Treatment for myiasis aims to remove the larvae from the body and involved tissue. Larvae are usually removed manually for cutaneous myiasis, followed by debridement and bandaging. Oral anthelminthic drugs like Ivermectin are required to treat cutaneous myiasis.^8^ In some cases of bladder myiasis, surgical intervention may be necessary, especially in the migratory form of the disease. Using 15% chloroform in olive or other oils, or even ether, demobilizes the larvae.

The common factors are the lack of sanitation, urinating in dirty toilets causing flies to sit or bite the perineal area during urination, not using a mosquito net, low immunity, female patients, and lower socioeconomic status.^14^ Myiasis can prevented by using pesticides and mosquito repellents. Ironing is an effective method for killing the eggs. Other preventive measures include wearing long‐sleeved clothes to cover the wounds and not sleeping outdoors.^15^ In our patients, lower socioeconomic status, diminished immunity due to prior radiation, and unsanitary conditions were the contributory factors.

Urinary infection, radiation cystitis, and tumor recurrence are the differential diagnoses of bladder myiasis. In cancer survival patients, any abnormal growth in any part of the body is an alarming sign and requires evaluation to confirm the diagnosis. A microscopic urine examination is needed to diagnose fulminant or advanced cases of bladder myiasis.

Direct visualization of the urinary bladder may require in doubtful cases of urinary myiasis. Urinary myiasis is a rare, uncommon finding, even for an experienced urologist who performs cystoscopies, so it is a limitation in diagnosing bladder myiasis. Diagnosing and identifying larvae under the microscope is also challenging.^16^

Incidences of cancer are increasing in the present era due to various reasons, like early diagnosis of malignancy. So, during the treatment of malignancy, the patient will undergo multiple treatments, which may develop poor hygiene, weight loss, and low immunity. These may require good hygiene, proper nutrition, and sanitation. This case is one of the crucial ones.

In this unique case, we speculate that the larvae infected the patient due to the dirty vaginal discharge. The anatomical and physiological characteristics of the female urethra, low immunity due to malignancy, and poor personal hygiene would have increased her likelihood of infection. Fortunately, the human body cannot complete the parasite life cycle. Therefore, patients recover when they improve their immunity and sanitary conditions.

## 4 CONCLUSION

Bladder myiasis is an infrequent finding, and this is one of the rare cases of post‐radiation bladder myiasis in the current literature. Regular follow‐up, clinical and radiological examination after treatment, and proper hygiene are the preventive measures for the best outcome in bladder myiasis. Haematuria, lower abdominal pain, and recurrent TI are presenting symptoms. Proactive and timely evaluation and diagnosis of bladder myiasis are vital to better outcomes.

## AUTHOR CONTRIBUTIONS

**Mahendra Kumar:** Conceptualization; data curation; formal analysis; methodology; project administration; resources; software; writing – original draft; writing – review and editing. **Sujit Saikia:** Conceptualization; investigation; supervision; writing – review and editing. **Anup Kumar Das:** Conceptualization; investigation; methodology; supervision; writing – review and editing. **Debabrata Barmon:** Conceptualization; methodology; project administration; supervision; writing – original draft; writing – review and editing. **Upasana Baruah:** Conceptualization; methodology; supervision; writing – original draft; writing – review and editing. **Dimpy Begum:** Conceptualization; methodology; supervision; writing – original draft; writing – review and editing. **Sopouassi V. Nicholas King:** Software; supervision.

## FUNDING INFORMATION

None.

## CONFLICT OF INTEREST STATEMENT

None.

## ETHICS STATEMENT

Not applicable.

## CONSENT

Written informed consent was obtained from the patient to publish this report in accordance with the journal's patient consent policy.

## Data Availability

The data that support the findings of this study are available on request from the corresponding author. The data are not publicly available due to privacy or ethical restrictions.
